# Impact of the Remission of Type 2 Diabetes on Cardiovascular Structure and Function, Exercise Capacity and Risk Profile: A Propensity Matched Analysis

**DOI:** 10.3390/jcdd10050191

**Published:** 2023-04-24

**Authors:** Joanna M. Bilak, Jian L. Yeo, Gaurav S. Gulsin, Anna-Marie Marsh, Manjit Sian, Abhishek Dattani, Sarah L. Ayton, Kelly S. Parke, Moira Bain, Wenjie Pang, Sherif Boulos, Tim G. St Pierre, Melanie J. Davies, Thomas Yates, Gerry P. McCann, Emer M. Brady

**Affiliations:** 1 Department of Cardiovascular Sciences, University of Leicester and the National Institute for Health Research (NIHR) Leicester Biomedical Research Centre, Leicester LE3 9QP, UK; jmb99@leicester.ac.uk (J.M.B.); jian.yeo@leicester.ac.uk (J.L.Y.); gg149@leicester.ac.uk (G.S.G.); anna-marie.marsh@uhl-tr.nhs.uk (A.-M.M.); ad530@leicester.ac.uk (A.D.); sa768@leicester.ac.uk (S.L.A.);; 2 Public and Patient Involvement Representative for National Institute for Health Research (NIHR) Leicester Biomedical Research Centre, Leicester LE3 9QP, UK; 3 Resonance Health Ltd., Burswood, WA 6100, Australia; 4 School of Physics, The University of Western Australia, Perth, WA 6009, Australia; 5 Diabetes Research Centre, NIHR Leicester Biomedical Research Centre, Leicester LE3 9QP, UK; melanie.davies@uhl-tr.nhs.uk (M.J.D.);

**Keywords:** diabetes remission, magnetic resonance imaging, cardiovascular reverse remodeling, risk factor control, type 2 diabetes, exercise capacity

## Abstract

Type 2 diabetes (T2D) confers a high risk of heart failure frequently with evidence of cardiovascular structural and functional abnormalities before symptom onset. The effects of remission of T2D on cardiovascular structure and function are unknown. The impact of the remission of T2D, beyond weight loss and glycaemia, on cardiovascular structure and function and exercise capacity is described. Adults with T2D without cardiovascular disease underwent multimodality cardiovascular imaging, cardiopulmonary exercise testing and cardiometabolic profiling. T2D remission cases (Glycated hemoglobin (HbA1c) < 6.5% without glucose-lowering therapy, ≥3 months) were propensity score matched 1:4 based on age, sex, ethnicity and time of exposure to those with active T2D (*n* = 100) with the nearest-neighbour method and 1:1 with non-T2D controls (*n* = 25). T2D remission was associated with a lower leptin–adiponectin ratio, hepatic steatosis and triglycerides, a trend towards greater exercise capacity and significantly lower minute ventilation/carbon dioxide production (VE/VCO2 slope) vs. active T2D (27.74 ± 3.95 vs. 30.52 ± 5.46, *p* < 0.0025). Evidence of concentric remodeling remained in T2D remission vs. controls (left ventricular mass/volume ratio 0.88 ± 0.10 vs. 0.80 ± 0.10, *p* < 0.025). T2D remission is associated with an improved metabolic risk profile and ventilatory response to exercise without concomitant improvements in cardiovascular structure or function. There is a requirement for continued attention to risk factor control for this important patient population.

## 1. Introduction

Type 2 diabetes mellitus (T2D) confers an excess risk of heart failure with a propensity towards preserved ejection fraction (HFpEF) [1]. T2D-related cardiovascular remodeling [2] (diabetic cardiomyopathy) presents early in the disease process and progresses insidiously. Exercise intolerance is an early symptom of heart failure [3] and a prognostic indicator in T2D [4]. The precise pathophysiological mechanisms underlying the exercise intolerance in HFpEF are not well understood and likely include a combination of cardiovascular (cardiac remodeling including left ventricular diastolic dysfunction, left atrial hypertrophy, arterial stiffness and microvascular dysfunction) and peripheral factors (sarcopenia and frailty) [5,6]. Short-term favourable improvements in cardiovascular remodeling and exercise tolerance following acute weight loss with a low-calorie meal replacement plan [7] or bariatric surgery [8] have been demonstrated, but such interventions are not feasible in all patients.

To date there remains a lack of evidence regarding predictors and medical outcomes including mortality, cardiovascular events and the functional capacity of the remission of T2D [9].

This was recently recognized by the American Diabetes Association who convened an international, multidisciplinary expert group to discuss current knowledge and build a consensus statement for the purpose of informing research and thus future clinical guidelines for people in remission for which there remains none to date. This expert group made a specific call for data on the impact of T2D remission beyond weight-loss and glycaemia [9].

In a recent cohort study, remission of T2D was associated with an improvement in the cardiovascular risk profile, but changes in cardiac structure and function or exercise capacity were not studied [10]. The aim of this study was to assess medium-term novel observations of the effects of the remission of T2D on cardiovascular structure and function and measures of exercise capacity, using the gold-standard techniques of MRI [11] and cardiopulmonary exercise testing [12], respectively, compared to people with active T2D of similar exposure time and to healthy controls. Specifically, these individuals achieved self-directed remission in the community, outside the confines of a clinical trial or medical intervention such as bariatric surgery. We have termed this ‘autonomous’ remission to acknowledge the lifestyle changes they engaged in since learning of their diagnosis.

## 2. Materials and Methods

Three hundred people with T2D and 45 healthy controls were prospectively recruited into the “Prevalence and determinants of subclinical cardiovascular dysfunction in adults with type 2 diabetes” study (PREDICT, NCT03132129) between May 2018 and March 2022 from across Leicester, Leicestershire and Rutland in the United Kingdom [13]. PREDICT is a prospective cross-sectional study with case-control. Upon recruitment, twenty-five participants were identified having achieved autonomous remission of T2D in the community through either self-directed diet and/or self-directed exercise without medical intervention. Remission of T2D was defined by a glycated hemoglobin (HbA1c) of <48 mmol/mol or 6.5% for ≥3 months in the absence of glucose-lowering therapy or other medical intervention [9]. To obtain estimates of the average effect of exposure to remission on the outcomes of interest, we employed propensity score matching, which is increasingly used in observational research [14]. To minimize bias, cases in remission were propensity score matched with a ratio of 1:4 based on age, sex, ethnicity and time of exposure to T2D to those in active T2D (*n* = 100) using the nearest-neighbour method [15]. Additionally, 25 non-T2D controls were propensity score matched on a ratio of 1:1, due to the availability of control participants, based on age, sex and ethnicity to those in T2D remission. In total, 150 participants were included in this analysis. The National Research Ethics Service granted approval (17/WM/0192). The study was conducted in accordance to the International Conference on Harmonisation GCP Guidelines (ICH GCP) with informed consent given prior to any data collection.

### 2.1. Assessments

Fasting plasma samples were assayed for renal and lipid profiles, full blood count, measures of glycaemia and cardiac enzymes in an accredited NHS Trust laboratory. Insulin and leptin were quantified by multiplex assay on a Luminex platform. Plasma adiponectin was analysed using a Quantikine ELISA assay (R&D Systems, Minneapolis, MN, USA).

Multi-parametric magnetic resonance imaging (MRI) was performed on a 3-Tesla MRI machine (Siemens, Erlangen, Germany) for the assessment of the left ventricular (LV) mass, volumes, adenosine stress and rest perfusion, myocardial extracellular volume (ECV) and late gadolinium enhancement [13] and for hepatic and pancreatic fat using a commercial volumetric fat fraction method (HepaFat^®^, Resonance Health Analysis Services, Burswood, Australia) [16]. Cardiac MRI images were analysed using CVi42 (Circle Cardiovascular Imaging, Calgary, AB, Canada) by two trained observers blinded to the clinical details. Cardiac computed tomography was performed for coronary calcium scoring, and epicardial adipose tissue was measured using a deep learning method (QFat, Cedars-Sinai Medical Center, Los Angeles, CA, USA). Myocardial perfusion reserve was derived as a ratio of stress to rest myocardial blood flow (MBF). For echocardiographic diastolic function, images were acquired and reported as per the American Society of Echocardiography guidelines by one of two accredited operators using an iE33 system with S5-1 transducer (Philips Medical Systems, Best, The Netherlands) to estimate left ventricular (LV) filling pressures (E/e’).

A one-minute incremental bicycle cardiopulmonary exercise test (CPET) was performed with expired gas analysis for peak VO2 and VE/VCO_2_ slope, derived from a regression line of minute ventilation and carbon dioxide production [17].

**Figure 1:**
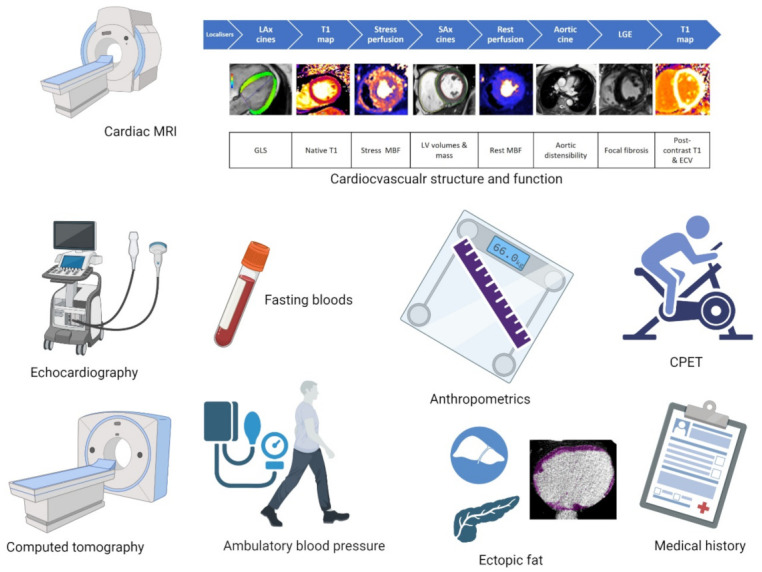
Study assessments conducted on the single day study visit. Abbreviations: MRI = magnetic resonance imaging, GLS = global longitudinal strain, MBF = myocardial blood flow, LV = Left ventricular, ECV = extracellular volume, CPET = cardiopulmonary exercise testing.

All data were collected on the same day (Figure 1).

### 2.2. Statistical Analysis

Descriptive statistics were used to characterize groups. The T2D remission group was compared to the active T2D group and then compared to non-T2D controls. Demographic, anthropometric and glycaemic indices were not tested for statistical significance given their expectedness in the context of the design of this study. For the more novel measures, an independent sample *t*-test, Mann–Whitney or Chi-square/Fisher’s exact tests were applied for comparisons, as appropriate. Statistical significance with adjustment for multiple testing (Bonferroni correction) was defined as *p* = 0.025, representing the adjusted *p*-value per group comparison (T2D remission vs. active T2D and T2D remission vs. non-T2D control). Statistical analysis was performed using IBM SPSS Statistics for Windows, Version 26.0 (Armonk, NY, USA: IBM Corp. Released 2019).

## 3. Results

The anthropometric, clinical and biochemical characteristics are provided by diabetes status in Table 1 with expected variance between groups for demographic, anthropometric and metabolic profiles. Average exposure to T2D was less than six years across both groups, and the duration of T2D remission was 4.2 ± 2.5 years. Only those with active T2D were taking glucose-lowering therapies, and there was comparable use of over- the-counter dietary supplements. Despite lower statin use in those in T2D remission, triglycerides were lower and HDL cholesterol significantly higher than in those with active T2D. Those with active T2D exhibited a more adverse inflammatory profile (higher leptin: adiponectin ratio). Further, even with normal HbA1c, there was evidence of impaired fasting glucose (>5.6 mmol/L) in more than 80% of those with T2D remission compared to 28% in non-T2D controls, with 22% exhibiting levels considered within the diabetes range.

There was a three-fold lower level of hepatic fat and a trend towards lower pancreatic fat (*p* = 0.106) and volume of epicardial adipose tissue (*p* = 0.104) in T2D remission compared to active T2D. All ectopic fat depots were comparable between those in remission and non-T2D controls. Differences in coronary calcium scores or echocardiographic measures of diastolic function were not statistically significant in either comparison.

**Figure 2:**
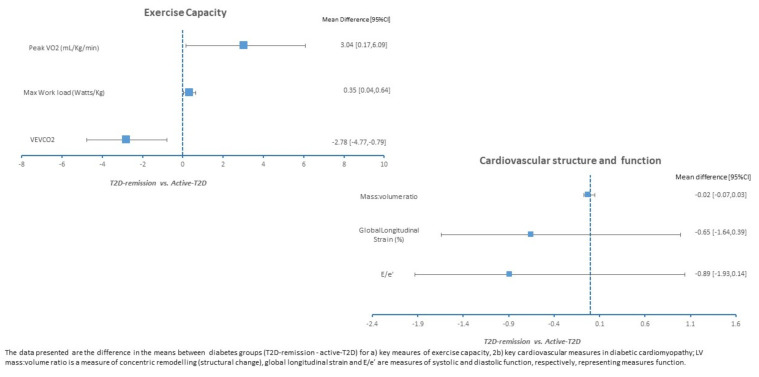
Difference in the mean value between groups for important exercise and cardiovascular measures for diabetic cardiomyopathy (T2D-remission—active-T2D).

Ambulatory 24 h blood pressures and anti-hypertensive medication use were similar between the T2D groups, and systolic blood pressure was significantly elevated in the T2D remission group versus controls. Cardiac biomarkers were within a normal range across groups. A graphical summary of the key exercise capacity and cardiovascular structure and function measures are presented in Figure 2, and all data are presented in Table 2. There were no clinically or statistically significant differences between the measures of cardiovascular structure or function between those in remission and those with active T2D. Consistent with this, concentric remodeling was increased in those in T2D remission compared to non-T2D controls (higher LV mass: volume ratio). MPR, a measure of microvascular function [18], and ECV did not significantly differ between the groups.

There was a trend towards higher peak VO_2_ under T2D remission compared to active T2D (*p* = 0.051) and a non-significant increase in the maximum workload *per kilogram*. The VE/VCO_2_ slope was significantly lower in the T2D remission group compared with the active T2D group and was comparable to that of the controls.

## 4. Discussion

This study adds important novel findings in response to the call for “*active observation of individuals experiencing a remission*” by the international diabetes remission panel [9]. We report novel observations, beyond that of glycaemia, with a specific focus on the cardiovascular structure, function and exercise capacity in people achieving autonomous remission of T2D outside of the rigor of a clinical trial or clinical intervention.

T2D is a multisystemic disorder, which adversely impacts the cardiovascular system, increasing the risk of heart failure as one of the most deleterious complications. Autonomous remission of T2D was associated with a more favorable cardiovascular risk profile, namely: lower circulating triglycerides, lower leptin–adiponectin ratio, better insulin homeostasis and higher HDL cholesterol levels compared to those with active T2D of the same duration. The importance of monitoring ectopic fat has become increasingly recognized and specifically, hepatic fat, in the wake of the twin-cycle hypothesis [19]. Hepatic steatosis was significantly lower in those in remission with a non-significant trend for a reduction in pancreatic fat. Further, there was a trend towards lower epicardial adipose volumes in those in remission compared to active T2D. Epicardial adipose tissue is associated with inflammation and cardiac fibrosis, is purported to play an important role in the pathophysiology of HFpEF, is associated with greater elevation in cardiac filling pressures and more severe pulmonary hypertension, culminating in poorer exercise capacity [20].

Exercise intolerance is highly prevalent in individuals living with T2D and can be the first sign of developing HFpEF [21]. We observed a trend towards better exercise capacity as measured by peak VO_2_ in T2D remission compared to active T2D. The VE/VCO_2_ slope corresponds to the ventilation increase in response to CO_2_ production, reflecting the ventilatory drive. It has been shown to be a prognostic marker in heart failure, with similar or higher accuracy than VO_2_ peak for predicting HF hospitalisation in HFpEF [22]. The VE/VCO_2_ slope was significantly lower in those in remission compared with active T2D and similar to the non-T2D controls.

Diabetic cardiomyopathy is a precursor of HFpEF and is typified by diastolic dysfunction, increased LV mass and reduced LV volumes [2]. Despite improvements in body composition, exercise capacity and cardiometabolic profile, T2D remission was not associated with any differences in structural or functional indices on cardiac MRI or echocardiography compared to those with active T2D. The LV mass/volume ratio, a key measure of concentric remodeling, was higher in those in remission compared to non-T2D controls. These findings suggest that achieving remission may not be associated with cardiac reverse remodeling, and this patient group may require further intervention to reduce its overall cardiovascular risk. A potential mechanism at play could be the downregulation of the protein Sirtuin 1, an NAD+-dependent deacetylase, for which there is significant variation in the expression and activity levels in response to exogenous and endogenous stimuli, with circulating levels being either advantageous or pathogenic [23]. Plasma levels have been reported to be lower in established cases of HFpEF [24], and it is considered a potential mediator of age-related cardiovascular diseases [25]. It is shown to play an important role in the development of cardiac hypertrophy, fibrosis and inflammation, all of which are pathophysiological mechanisms underlying HFpEF, likely through its link to the renin–angiotensin aldosterone system [24,26]. Collectively, this needs to be confirmed in further longitudinal and interventional studies. MPR, which we have previously shown to be independently associated with exercise capacity in T2D [27], was not significantly different between groups. This finding may be attributed to microvascular dysfunction being a late phenomenon in the pathophysiology of T2D-related HFpEF [28].

These results indicate that whilst there are clear benefits, outside of glycaemic control and weightloss, to achieving autonomous remission of T2D, the patient group does not phenotypically align with matched non-T2D controls. Notably, the change in the cardiovascular risk profile was not similarly associated with the manifested structural and functional changes in the heart. Perhaps this reflects the debated ‘*metabolic memory*’ phenomenon [29], whereby, a hyperglycaemic environment activates a cascade of events resulting in the continual generation of oxidative stress irrespective of a return to normoglycaemic conditions seen in diabetes remission. This in turn exerts persistent detrimental effects on the cardiovascular system, resulting in diabetes-related complications independent of glycaemia [30]. Mitochondrial dysfunction is central to the activation of this cascade with the overproduction of superoxide anions following mitochondrial protein glycation, i.e., advanced glycated end products (AGEs), due to the excess glucose in the hyperglycaemic environment. Mitochondrial superoxide overproduction then activates four key pathways in a glycaemic-independent manner; protein kinase C, the polyol pathway, the hexosamine pathway flux and increased AGE formation, collectively resulting in the further generation of oxygen radicals, cellular injury and the maintenance of the pathogenic pathways of diabetes complications. In terms of cardiac insult, Yao et al., albeit in a mouse model, recently described endothelial cell “*metabolic memory*” and cardiac dysfunction in diabetes. The “*metabolic memory*” of the endothelial cells resulted in the upregulation of extracellular matrix-related and pro-fibrotic genes causing cardiac perivascular fibrosis and subsequent cardiac dysfunction [31] under normoglycaemic conditions.

Moreover, whilst the T2D remission group met the recognized definition for remission, 82% had evidence of impaired fasting glucose. Therefore, these data may more likely support the recent coining of the physiological state of ‘*post-diabetes*’ [19] and/or requirement for further categorization of remission [32] with the inclusion of plasma glucose levels. In either case, continued attention to risk factor control is required since the long-term outcomes remain unknown, in addition to the residual cardiovascular risk observed in patients who achieve remission through bariatric surgery [33]. Further long-term data are needed to assess the risk of heart failure in those who achieve remission.

### Strengths and Limitations

The major strength of this work is the detailed phenotyping of the cohort including state-of-the-art MRI imaging techniques to characterize cardiovascular structure and function within the multi-ethnic cohort. Participants were prospectively recruited, and image analysis and biochemistry were conducted blinded to participant details and other results. We employed a propensity score matched design 1:4 for remission and active disease groups and 1:1 for remission and control to improve analytical power; however, the key limitation of this study is the small sample size. Further, whilst there was fair representation of females, the total sample was over represented by White Europeans; therefore, these results need confirmation in a larger study with greater ethnic variation.

## 5. Conclusions

T2D remission is associated with improved metabolic risk profile and ventilatory response to exercise. However, T2D remission is not associated with concomitant improvements in cardiovascular structure or function; rather, there is evidence of cardiac concentric remodeling. This important and growing patient population requires continued attention for risk factor control.

## Figures and Tables

**Table 1:** Participant characteristics by diabetes classification.

	T2D Remission (*n* = 25)	Active T2D (*n* = 100)	Controls (*n* = 25)
**Demographic, Anthropometric and Glycaemic Characteristics**			
Age (years)	62.1 ± 8.9	62.1 ± 7.0	59.4 ±7.3
Male (%)	15 (60)	63 (63)	13 (52)
White Ethnicity (%)	20 (80)	80 (80)	13 (52)
Smoking, Never (%)	14 (56)	53 (53)	17 (68)
Height (cm)	169.5 ± 9.8	170.49 ± 9.01	170.4 ± 9.2
Weight (kg)	78.3 ± 17.3	93.2 ± 19.1	79.4 ± 15.2
BMI (kg/m^2^)	27.1 ± 4.7	32.1 ± 6.4	27.3 ± 3.9
Weight–height ratio (kg/cm)	0.46 ± 0.09	0.55 ± 0.11	0.46 ± 0.07
Waist circumference (cm)	99.1 ± 13.1	109.9 ± 11.1	97.0 ± 11.8
Waist–hip ratio	0.93 ± 0.07	0.97 ± 0.06	0.91 ± 0.07
Years since T2D diagnosis	5.7 ±4.4	5.6 ± 4.2	-
Remission (years)	4.2 ± 2.5	-	-
**Medication**			
Number of antihypertensives ^$^			
0	17 (68)	49 (49)	23 (92)
1	3 (12)	25 (25)	2 (8)
≥2	5 (20)	26 (26)	0 (0)
Statin (%)	9 (36)	65 (65)	6 (24)
Dietary supplement (%)	6 (24)	28 (28)	12 (48)
**24 h ambulatory blood pressure and heart rate**			
Systolic (mmHg)	127.3 ± 11.2	127.6 ± 12.7	118.7 ± 11.3 ^†^
Diastolic (mmHg)	76.7 ± 8.3	75.2 ± 7.1	73.0 ± 6.6
Heart rate (bpm)	73.0 ± 10.8	75.9 ± 10.3	68.1 ± 6.9
**Biochemistry**			
eGFR (mL/min/1.72 m^2^)	80 (70, 89)	78 (74, 84)	79 (73, 83)
NT-proBNP (pg/mL)	62 (4, 111)	59 (41.5, 93.0)	48 (39, 99)
Hs-Trop I (ng/L)	5.80 (3.20, 7.60)	3.35 (2.70, 5.30)	4.20 (3.05, 4.65)
Leptin (ng/mL)	6.30 (4.15, 12.60)	17.40 (12.14, 29.43) *	9.52 (4.53, 21.77)
Adiponectin (g/mL)	9.39 (8.02, 13.21)	8.02 (6.96, 9.74)	10.42 (8.11, 11.90)
Leptin:Adiponectin Ratio (LAR)	0.75 (0.37, 1.50)	2.24 (1.43, 4.10) *	0.89 (0.46, 2.07)
HbA1C (%)	5.84 ± 0.44	7.39 ± 1.09	5.49 ± 0.32
HbA1c (mmol/mol)	40.24 ± 4.95	57.24 ± 12.00	36.46 ± 3.49
Fasting glucose (mmol/L)	6.15 ± 0.82	8.30 ± 2.05	5.21 ± 0.56
IFG *pre-diabetes range* (5.6–6.9 mmol/l, (*n* (%))	15 (60)	17 (17) *	7 (28) ^†^
IFG *T2D range* (> 7.0 mmol/l, (*n* (%))	5 (22)	75 (75) *	0 (0) ^†^
Insulin μIU/mL	12.99 (11.56, 16.39)	18.56 (11.21, 26.70)	6.43 (4.89, 10.50)
HOMA-IR	3.38 (2.88, 4.61)	6.84 (4.20, 11.11)	1.64 (1.08, 2.50)
Cholesterol (mmol/L)	4.90 ± 1.33	4.43 ± 1.01	5.40 ± 1.08
HDL cholesterol (mmol/L)	1.40 (1.20, 1.90)	1.20 (1.00, 1.50) *	1.65 (1.23, 2.08)
LDL cholesterol (mmol/L)	2.62 ± 1.02	2.32 ± 0.81	3.18 ± 0.97
Triglycerides (mmol/L)	1.45 (0.96, 1.74)	1.69 (1.3, 2.2) *	1.18 (0.98, 1.45)

Data presented are the mean ± standard deviation or median and interquartile range (25th and 75th quartiles). Abbreviations: T2D = type 2 diabetes; BMI = body mass index; eGFR = estimated glomerular filtration rate; NT-proBNP = brain natriuretic peptide; Hs-trop = high sensitivity troponin; HbA1C = glycosylated haemoglobin A1C; IFG = impaired fasting glucose (fasting glucose only available for 97 active T2D); HOMA-IR = homeostatic index of insulin resistance; HDL = high density lipoprotein; LDL = low density lipoprotein. NB: Plasma insulin available for *n* = 59 active, *n* = 22 non-T2D control and *n* = 12 remission; leptin and adiponectin available for *n*= 61 active, *n* = 22 non-T2D controls and *n* = 12 remission. Bonferroni adjusted level of significance *p* = 0.025. * Statically significant difference between T2D remission and active T2D, **^†^** statistically significant difference between T2D remission and non-T2D controls. ^$^ Includes the use of any class of antihypertension medication: ACE inhibitors, angiotensin receptor blockers, beta-blockers, calcium channel blockers and/or diuretics.

**Table 2:** Results of multimodality cardiovascular phenotyping.

	T2D Remission (*n* = 25)	Active T2D (*n* = 100)	Controls (*n* = 25)
**Echocardiography**			
E/A ratio	0.94 ± 0.31	0.91 ± 0.21	1.04 ± 0.19
E/e’	8.1 ± 2.2	9.0 ± 2.0	8.7 ± 1.5
**Magnetic resonance imaging**			
Hepatic fat (%)	3.20 (2.20, 7.33)	9.40 (4.80, 15.20) *	2.70 (1.90, 3.70)
Pancreatic fat (%)	3.80 (3.15, 6.45)	6.20 (3.60, 9.30)	3.30 (1.80, 5.90)
LV mass/height (g/m)	0.66 ± 0.13	0.71 ± 0.16	0.67 ± 0.14
LVEDV/height (ml/m)	0.77 ± 0.14	0.80 ± 0.19	0.84 ± 0.14
LV mass: volume	0.88 ± 0.10	0.90 ± 0.13	0.80 ± 0.10 ^†^
LV EF (%)	66.1 ± 7.3	66.7± 6.8	67.5 ± 3.9
LV GCS (%)	18.8 ± 2.4	19.1 ± 2.3	19.5 ± 1.8
LV GLS (%)	15.8 ± 2.3	16.4 ± 2.2	17.4 ± 1.9
ECV (%)	27.0 ± 2.7	27.0 ± 2.5	26.0 ± 1.7
LGE infarction (*n* (%))	10 (40)	28 (28)	14 (56)
Myocardial perfusion reserve	2.84 ± 0.75	2.88 ± 0.80	3.13 ± 0.87
**Cardiac computed tomography**			
Calcium score ^$^ <1	13 (56.5)	30 (30.6)	13 (52.0)
Calcium score ^$^ 1–100	4 (17.4)	37 (37.7)	4 (16.0)
Calcium score ^$^ >100	6 (26.1)	31 (31.6)	8 (32.0)
Epicardial adipose volume (mL)	111.36 ± 67.38	144.22 ± 54.84	89.41 ± 40.48
**Cardiopulmonary exercise testing**			
Max. workload (watts)	127.68 ± 54.59	124.73 ± 46.68	152.28 ± 53.46
Max. workload/weight (watts/Kg)	1.65 ± 0.69	1.30 ± 0.54	1.91 ± 0.55
Peak VO_2_ (L/min)	1.72 ± 0.60	1.74 ± 0.51	1.89 ± 0.55
Peak VO_2_ (mL/kg/min)	22.20 ± 7.05	19.16 ± 4.90	23.94 ± 5.55
VE/VCO_2_ slope	27.74 ± 3.95	30.52 ± 5.46 *	27.09 ± 4.57
Max. exercise heart rate (bpm)	155.8 ± 18.6	150.8 ± 18.2	157.4 ± 15.0
Respiratory exchange ratio peak	1.06 ± 0.06	1.06 ± 0.08	1.10 ± 0.07

Data presented are the mean ± standard deviation or median and interquartile range (25th and 75th quartiles). Abbreviations: T2D = type 2 diabetes; EDV= end diastolic volume; E/e’—early mitral filling to early mitral annular velocity; LV = left ventricle, EF = ejection fraction; GCS = global circumferential strain, GLS = global longitudinal strain, ESV = end systolic volume; LGE= late gadolinium enhancement; LV = left ventricle/left ventricular. Bonferroni adjusted level of significance *p* = 0.025. ^$^ Agatston units. * Statically significant difference between T2D remission and active T2D, **^†^** statistically significant difference between T2D remission and non-T2D controls.

## Data Availability

Data available upon request to the corresponding author.

## Abbreviations

CPET—cardiopulmonary exercise testing; HFpEF: heart failure with preserved ejection fraction; MRI: magnetic resonance imaging.
